# Extended antibody-framework-to-antigen distance observed exclusively with broad HIV-1-neutralizing antibodies recognizing glycan-dense surfaces

**DOI:** 10.1038/s41467-021-26579-z

**Published:** 2021-11-09

**Authors:** Myungjin Lee, Anita Changela, Jason Gorman, Reda Rawi, Tatsiana Bylund, Cara W. Chao, Bob C. Lin, Mark K. Louder, Adam S. Olia, Baoshan Zhang, Nicole A. Doria-Rose, Susan Zolla-Pazner, Lawrence Shapiro, Gwo-Yu Chuang, Peter D. Kwong

**Affiliations:** 1 Vaccine Research Center, NIAID, National Institutes of Health, Bethesda, MD 20892 USA; 2 Department of Medicine and Department of Microbiology, Icahn School of Medicine at Mount Sinai, New York, NY 10029 USA; 3 Department of Biochemistry and Molecular Biophysics, Columbia University, New York, NY 10032 USA

**Keywords:** Structural biology, Classification and taxonomy, Cryoelectron microscopy

## Abstract

Antibody-Framework-to-Antigen Distance (AFAD) – the distance between the body of an antibody and a protein antigen – is an important parameter governing antibody recognition. Here, we quantify AFAD for ~2,000 non-redundant antibody-protein-antigen complexes in the Protein Data Bank. AFADs showed a gaussian distribution with mean of 16.3 Å and standard deviation (σ) of 2.4 Å. Notably, antibody-antigen complexes with extended AFADs (>3σ) were exclusively human immunodeficiency virus-type 1 (HIV-1)-neutralizing antibodies. High correlation (R^2^ = 0.8110) was observed between AFADs and glycan coverage, as assessed by molecular dynamics simulations of the HIV-1-envelope trimer. Especially long AFADs were observed for antibodies targeting the glycosylated trimer apex, and we tested the impact of introducing an apex-glycan hole (N160K); the cryo-EM structure of the glycan hole-targeting HIV-1-neutralizing antibody 2909 in complex with an N160K-envelope trimer revealed a substantially shorter AFAD. Overall, extended AFADs exclusively recognized densely glycosylated surfaces, with the introduction of a glycan hole enabling closer recognition.

## Abstract (web summary)

Here, the authors analyse the distance between the body of an antibody and a protein antigen denoted as the Antibody-Framework-to-Antigen Distance (AFAD) for about 2000 non-redundant antibody-protein antigen complexes in the Protein Data Bank. They observe that antibodies with exceptionally long AFADs were all broad HIV-1-neutralizing antibodies that targeted densely glycosylated regions on the HIV-1-envelope trimer. The connection between long AFAD and dense glycan was further validated by the cryo-EM structure of antibody 2909 recognizing a glycan hole and by glycan shielding analyses based on molecular dynamics simulations.

## Introduction

Antibody recognition of protein surfaces forms the basis of the humoral immune response. Such recognition is a subset of more general protein–protein interactions, and substantial analysis has been carried out to understand the fundamental properties that characterize a protein–protein interface, including the size and shape of the interface, the complementarity between interacting surfaces, the propensity of residues to form interfaces, the hydrophobicity–polarity of interfaces, the amount of interfacial solvent, the segmentation and secondary structure of interfacing regions, and the conformational alterations that occur upon complex formation^1,2^.

With antibodies, protein recognition is encoded by recombination and developed through immune processes of selection and maturation^3–5^. These immune processes enable the development of high-affinity recognition against virtually all protein antigens, through elaborations of six complementarity determining regions (CDRs), three on the antibody-heavy chain and three on the antibody-light chain, which comprise the antibody-recognition surface^6^. Because the highly variable CDRs extend from β-sheets that comprise the conserved body of the antibody, it is possible to analyze properties of the conserved body of the antibody in the context of overall antibody-protein recognition.

Here we explore an important parameter governing antibody recognition, Antibody-Framework-to-Antigen Distance (AFAD)—the distance between the conserved body of the antibody and the recognized protein antigen—and quantify AFAD for 1879 non-redundant antibody-protein antigen complexes in the Protein Data Bank^7^. To provide insight into results, we performed molecular dynamics (MD) simulations on glycosylated human immunodeficiency virus-type 1 (HIV-1) envelope (Env) structures and determined the cryo-EM structure of an HIV-1 Env trimer in complex with monoclonal antibody 2909, which recognizes a glycan hole at the trimer apex. We found long AFADs to be exclusive to broadly neutralizing antibodies targeting HIV and their AFADs to correlate with density of glycan coverage. Overall, the results provide insight into the requirements for antibody recognition of highly glycosylated antigens.

## Results

### Analysis of AFAD for all antibody complexes in the PDB

**Fig. 1:**
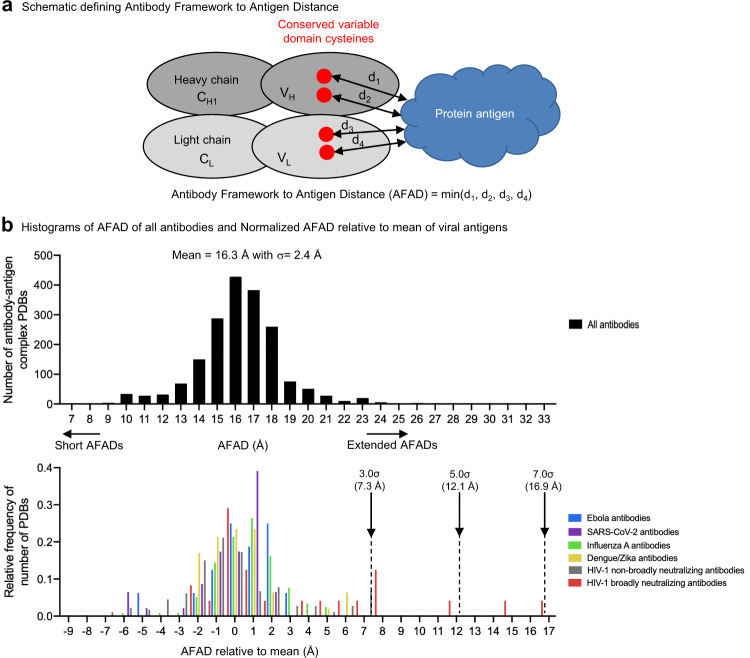
Analysis of Antibody-Framework-to-Antigen Distance (AFAD) for all antibody complexes in the PDB reveals outliers with extended recognition distances to be exclusively HIV-1 broadly neutralizing antibodies. **a** Schematic of AFAD calculated from the shortest protein antigen atom-distance to each of the four conserved variable domain cysteines (red). Only protein atoms were used for calculations, excluding distances for cofactors, glycans, and other non-protein atoms. **b** Histogram of AFAD for *n* = 1879 antibody complexes in the PDB (black). Normalized AFADs relative to mean are also shown for Ebola virus (blue), SARS-CoV-2 (purple), influenza A virus (green), Dengue/Zika virus (yellow), human immunodeficiency virus-1 (HIV-1) non-broadly neutralizing antibodies (gray), and broadly neutralizing antibodies targeting HIV-1 (red). AFAD relative to mean for *n* = 1879 PDB complexes formed a normal distribution as mean values 16.3 +/− 2.4 Å SD. Select HIV-1 broad neutralizing antibody-recognition distances can be extended by more than 3σ longer. AFADs in the bottom plot are normalized relative to mean (16.3 Å). See also Supplementary Table 1.

We defined AFAD as the closest distance between any heavy atom on the antigen and any of the heavy atoms on the four conserved cysteines on the variable domain (Fv)—residues C22 and C92 on the heavy chain, and C23 and C88 on the light chain—of the antibody (Fig. 1a, see “Methods” for details). Using this definition, we calculated AFAD for 1879 non-redundant antibody–antigen complexes from the Protein Data Bank^7^. We observed AFADs to form a Gaussian distribution, with a mean distance from cysteines of 16.3 Å and a standard deviation (σ) of 2.4 Å (Fig. 1b).

Short AFADs (~3σ below the mean) were observed for ligands that recognize the side of the antibody (Supplementary Fig. 1a left), with a peptide that penetrated into the CDR cleft (Supplementary Fig. 1a middle), or for ligands with large interactive surfaces that extended to the side of the antibody (Supplementary Fig. 1a right). Average AFADs were observed with both peptides and proteins that utilized CDRs for recognition and did not penetrate through or drape around CDRs (Supplementary Fig. 1b). Extended AFADs (~3σ above the mean) were observed for antibodies binding highly glycosylated surfaces, especially those on the HIV-1 Env glycoprotein trimer (Supplementary Fig. 1c, Supplementary Table 1).

We compared the AFADs of 23 HIV-1 Env targeting broadly neutralizing antibodies, each representing a unique antibody class^8^, and observed 8 of the 23 antibodies to have AFADs longer than the mean of the general distribution by 3σ (7.3 Å) (Fig. 1b). Notably, none of the antibody–antigen complexes for Ebola virus glycoprotein, SARS-CoV-2 spike, influenza A virus hemagglutinin, Dengue/Zika envelope exhibited entries with such high levels of AFAD (Fig. 1b) even though antibodies against these pathogens (except for SARS-CoV-2) were found in the top 50 for extended AFADs relative to the mean (Supplementary Table 1). Only a subset of the top 50 was significantly different from the mean (AFAD > 3σ), corresponding to the top 10 in Supplementary Table 1, which target densely glycosylated epitopes.

The 1879 PDB collection is composed of 823 viral pathogens and 1056 non-viral pathogens, with viral pathogens representing slightly less than half of the total PDB collection (Supplementary Fig. 2a). We evaluated the distribution of each viral antigen–antibody complex (e.g., HIV-1 non-broadly neutralizing, HIV-1 broadly neutralizing, and antibodies targeting influenza A virus, hepatitis C virus, Dengue/Zika virus, SARS-CoV-2, and Ebola virus) in Supplementary Fig. 2b and found HIV-1 antibody complexes (broadly neutralizing and non-broadly neutralizing combined) to dominate in the viral antigen dataset.

To examine a potential systematic bias toward observing longer AFADs with certain structure determination methods, we separated the entire PDB dataset by X-ray crystal structures and electron microscopy (EM) structures and analyzed the AFADs. The AFADs calculated from crystal structures (Supplementary Fig. 2c) and EM structures (Supplementary Fig. 2d) distribute in a similar manner and follow the total PDB collection distribution, suggesting there is no systematic bias toward observing longer AFADs with certain structure determination methods. This could not be factored when determining “deviation from the mean”. The deviation from the mean by what we observed is thoroughly from the shorter/longer distance of antibody–antigen recognition.

We calculated CDR H3/L3 lengths of all antibodies in the PDB dataset and show the distribution in Supplementary Fig. 2e and Supplementary Fig. 2f. The CDR H3 length distribution followed a Gaussian shape similar to AFAD distribution, whereas CDR L3 length showed a narrow distribution centered on 9 amino acids. We analyzed the correlation between AFAD and CDR H3 or CDR L3 length for 1879 non-redundant antibody-protein antigen complexes and found low correlations for CDR H3 length (*R*^2^ = 0.1652) (Supplementary Fig. 2g) and for CDR L3 length (*R*^2^ = 0.0086) (Supplementary Fig. 2h). However, the correlation between AFAD and CDR H3 length was higher (*R*^2^ = 0.4593) when analyzing antibodies with long CDR H3 (>24 amino acids) (Supplementary Fig. 2i).

### Only select categories of HIV-1 broadly neutralizing antibodies have extended AFADs relative to mean

**Fig. 2:**
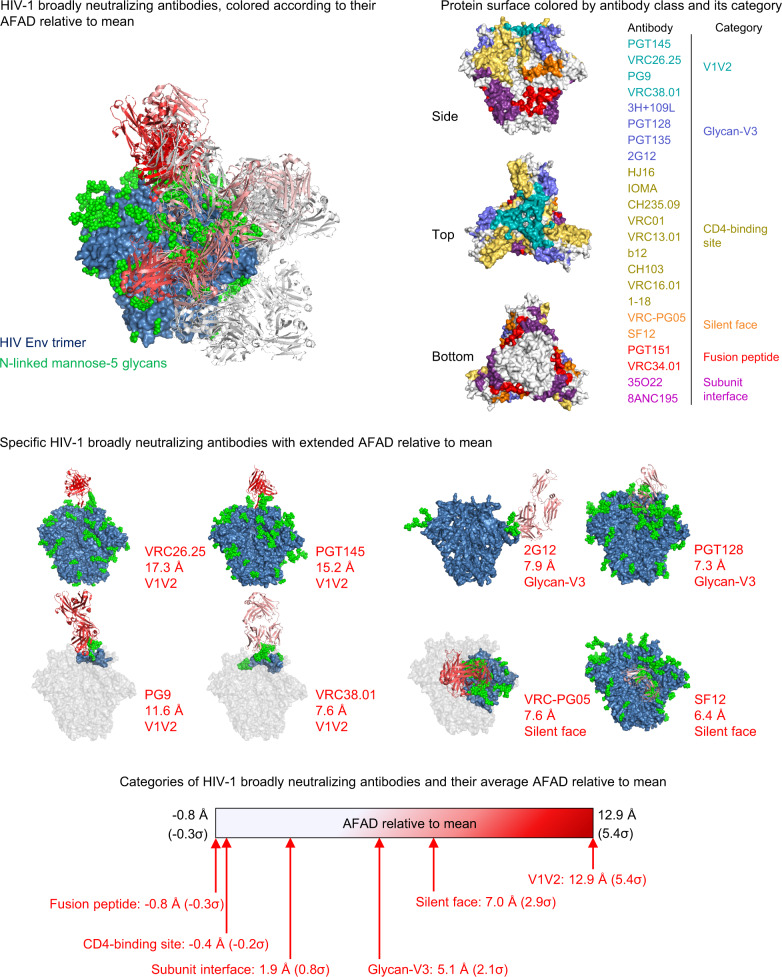
Only select categories of HIV-1 broadly neutralizing antibodies have extended AFADs relative to mean. HIV-1 broadly neutralizing antibodies, colored according to their AFAD relative to mean and the associated scale is shown at the bottom. Protein surface of Env trimer colored by epitope category (top right). See also Supplementary Table 2. Average AFAD relative to mean for classes of HIV-1 broadly neutralizing antibodies. The antigens are colored in blue if structures are available, otherwise in gray (middle).

Examining the AFAD of HIV-1 antibodies based on their epitope categories (V1V2, glycan-V3, CD4-binding site, silent face, fusion peptide, subunit interface)^8^, we observed the V1V2 class broadly neutralizing antibodies to possess the longest AFAD relative to mean on average (12.9 Å), followed by silent face antibodies (7.0 Å), and glycan-V3 antibodies (5.1 Å) (Fig. 2). Specifically, CAP256-VRC26.25 possessed the longest AFAD relative to mean (17.3 Å), followed by PGT145 (15.2 Å), PG9 (11.6 Å), 2G12 (7.9 Å), VRC-PG05 (7.6 Å), VRC38.01 (7.6 Å), PGT128 (7.3 Å), and SF12 (6.4 Å).

### Extended AFADs are observed with antibodies recognizing densely glycosylated regions

**Fig. 3:**
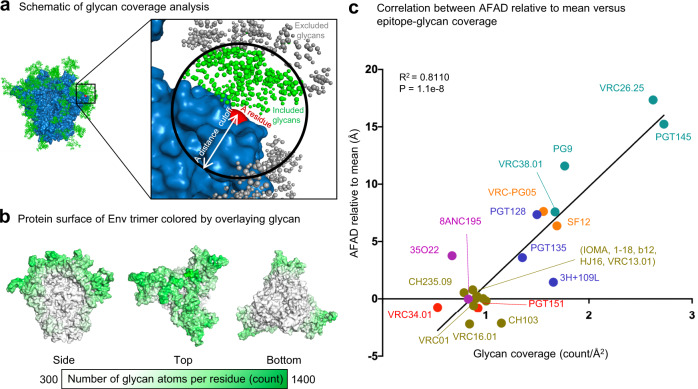
AFAD correlated significantly with epitope-glycan coverage. **a** Schematic figure for defining associated glycan atoms per residue. Glycan atoms within a cutoff are counted as number of glycan atoms per residue (green) except those are blocked by protein region (gray). Glycans outside of the cutoff are excluded in the analysis (gray). The protein surface is colored in blue and the residue of interest on the surface is colored in red. **b** Protein surface of BG505 WT Env trimer colored by number of overlaying glycan atoms. Number of glycan atoms overlaying each exposed protein surface residue was calculated by the algorithm described in (**a**) and averaged over 500 ns molecular dynamics simulations of fully glycosylated HIV-1 BG505 Env trimer. **c** Correlation of AFAD relative to mean versus glycan coverage overlaying antibody epitopes of BG505 WT. A simple linear regression is calculated with *n* = 22 antibodies. 2G12 is not presented in the plot due to absence of protein epitope residues. The antibody epitopes are colored as shown in Fig. 2 top right panel.

To examine the relationship between AFADs and glycan coverage on HIV-1 Env surface, we performed MD simulations of a BG505 wild-type (WT) Env trimer and quantified the number of glycans associated with each residue over the course of simulations (Fig. 3a). We observed that V1V2, glycan-V3, and silent face regions are the most densely glycosylated (colored in green to dark green), while the rest of the regions are covered with less glycans (in light green to white) (Fig. 3b).

We also found that antibodies with long AFADs recognized regions of the Env trimer with dense glycan shield coverage, whereas antibodies with short AFADs recognized regions of the Env trimer with sparse glycan coverage. A high correlation (*R*^2^ = 0.8110, *p* < 0.0001) was observed between AFAD relative to mean and glycan coverage for 23 classes of HIV-1 broadly neutralizing antibodies (Fig. 3c). Densely glycosylated areas such as V1V2, silent face, and glycan-V3 regions were observed to have glycan coverage beyond 1.39 counts/Å^2^ with equal or longer than 1.45 Å extended AFADs relative to mean, whereas the CD4-binding site, fusion peptide, and subunit interface had glycan coverages of lower than 1.39 counts/Å^2^, with shorter than 1.45 Å to negative AFADs relative to mean (except 35O22) (Supplementary Table 2).

We also examined the correlation between AFADs relative to mean and various antigen–antibody interaction properties (e.g., CDR H3 paratope surface area percentage, epitope total/protein surface area, CDR H3 length, epitope-glycan percentage, neutralization potency, epitope conservation, paratope surface area, somatic hypermutation, neutralization breadth) (Supplementary Fig. 3). While statistically significant, the correlation values were found to be less than that of glycan coverage versus AFAD relative to the mean. Notably, the paratope surface area percentage of CDR H3 was found to have the second strongest correlation (*R*^2^ = 0.6420) after AFAD versus glycan coverage, suggesting that the paratope of antibodies with long AFADs are dominated by CDR H3 surface area. The CDR H3 contact with antigen is mostly related to long AFAD while other CDRs are interacting with protruding glycan residues.

In addition, we calculated the correlation of glycan coverage with the same set of properties, and similarly, none showed a correlation as high as the correlation between AFAD relative to mean and glycan coverage (Supplementary Fig. 4).

When analyzing antibodies with long AFADs or dense epitope-glycan coverage (above the average) only, the correlation between AFADs and glycan coverage or CDR H3 length was similar or stronger than that observed with the full set of HIV-1 broadly neutralizing antibodies (*R*^2^ = 0.7444, 0.5545, respectively) (Supplementary Fig. 5a, b). Also, the correlation between AFAD and glycan coverage when epitope-glycan coverage is above average (Supplementary Fig. 5a) was found to be the same as glycan coverage versus CDR H3 (Supplementary Fig. 5c). Therefore, AFAD relates to glycan coverage (*R*^2^ = 0.7444) slightly stronger than the CDR H3 when CDR H3 is long (*R*^2^ = 0.5545) (Supplementary Fig. 5a and b).

### Structure of N160K glycan-hole recognizing antibody 2909 with Env trimer reveals similar recognition to PGT145, but with ~13 Å closer approach

**Fig. 4:**
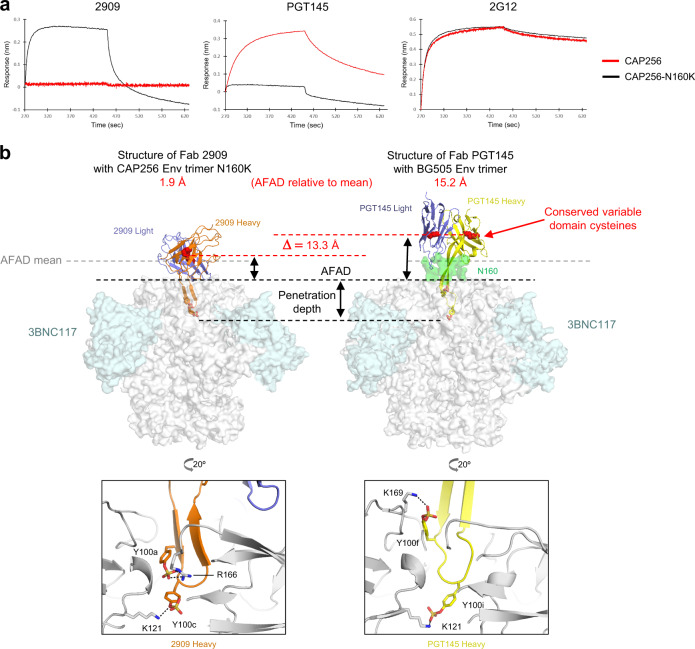
Structure of N160K glycan-hole recognizing antibody 2909 with Env trimer reveals similar recognition to PGT145, but with ~13 Å closer approach. **a** CAP256.wk34.c80 SOSIP.RnS2 Env trimer variants containing the native N160 glycan site (CAP256-WT) or the N160K mutation (CAP256-N160K) were assessed for binding to 2909, PGT145, and 2G12 antibodies using biolayer interferometry. **b** Cryo-EM structure of CAP256-N160K Env trimer (surface representation in gray) in complex with the Fabs from antibodies 2909 and 3BNC117; inset shows CDR H3 region of 2909 penetrating the apex of the trimer with two sulfated tyrosines, Tys100c shown interacting with K121; CDR H3 length is 21 in Kabat (left). Cryo-EM structure of BG505 Env trimer (surface representation in gray) in complex with the Fabs from antibodies PGT145 and 3BNC117 (PDB: 5V8L); inset shows CDR H3 region of PGT145 penetrating the apex of the trimer with two sulfated tyrosines, Tys100i shown interacting with K121. CDR H3 length is 31 in Kabat (right). See also Supplementary Fig. 6 and Supplementary Table 3.

We next investigated the effect of introducing a glycan hole on the HIV-1 Env trimer on AFAD. Human antibody 2909 is a strain-specific, HIV-1 neutralizing antibody targeting a quaternary V1V2 apex epitope containing a rare N160K substitution that removes a glycan^9–11^. To evaluate 2909 interactions with Env trimer containing an N160K glycan hole, we produced an N160K variant of the prefusion-closed stabilized CAP256.wk34.c80 SOSIP.RnS2 Env trimer^12^. Biolayer interferometry analysis confirmed 2909 binding to the CAP256-N160K glycan-knockout variant and not to CAP256 Env trimer containing the N160 glycan while the N160K mutation abolished recognition by PGT145 (Fig. 4a).

To provide a structural basis for 2909 recognition of the N160K glycan hole, we determined the 3.91 Å cryo-EM structure of the antigen-binding fragment (Fab) of 2909 in complex with the CAP256.wk34.c80 SOSIP.RnS2.N160K trimer bound to 3BNC117 Fab (Fig. 4b, Supplementary Fig. 6 and Supplementary Table 3). 3BNC117 was used to purify the trimer, to increase the mass and orientation distribution of particles for cryo-EM analysis, and to provide an analogous comparison to the PGT145-Env complex bound by 3BNC117. The CDR H3 of 2909 formed an extended hairpin, with sulfated tyrosine at its tip, highly similar to the previously determined ligand-free monoclonal antibody structure^13–15^. This extended hairpin inserted into the hole at the trimer apex to interact electrostatically with Lys121, in a manner similar to antibody PGT145^16,17^, which also possesses a CDR H3 which forms an extended hairpin, with sulfated tyrosine at its tip^13,18^ (Fig. 4b). Notably, the distance of the antibody 2909 from the N160K Env trimer was 13.3 Å closer than the distance of antibody PGT145 from the WT trimer.

### Antibody 2909 targeting the glycan hole of the N160K Env trimer induces a shorter AFAD relative to the mean but a larger protein epitope surface area compared to antibody PGT145

**Fig. 5:**
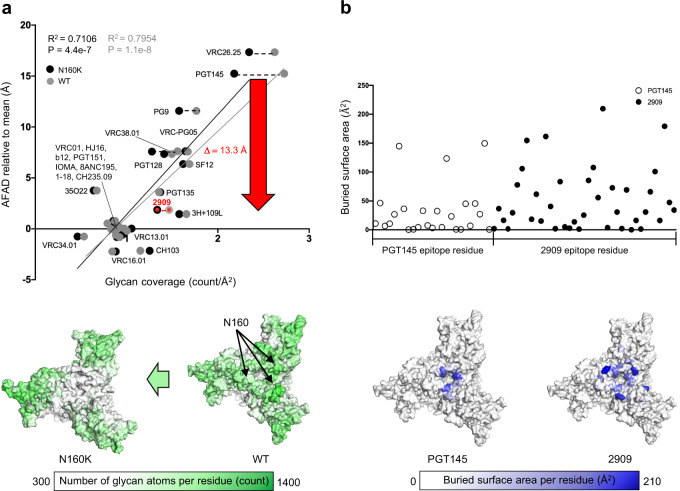
Antibody 2909 targeting to the glycan hole of N160K Env trimer induces shorter AFAD relative to mean but larger surface area compared to antibody PGT145. **a** Plot of AFAD relative to mean versus epitope-glycan coverage overlaying antibody epitopes of BG505 WT (dotted in gray) and BG505 N160K (dotted in black). Simple linear regressions are calculated with *n* = 23 antibodies on both WT and N160K. 2G12 is not presented in the plot due to the absence of protein epitope residues. 2909 is added to compare the difference between WT and N160K. The dash lines are plotted to the antibody epitopes that have the difference of glycan coverage higher than 0.2 (counts/Å^2^) between WT and N160K (top). The protein surface of BG505 WT (right) and N160K (left) Env trimer colored by number of overlaying glycan atoms (bottom). The red arrow indicates the difference of AFAD between PGT145 and 2909 which is 13.3 Å. **b** The buried surface area of each epitope residue for PGT145 and 2909 (top). The protein surface of epitope residues of PGT145 and 2909 are colored according to their buried surface area (bottom). See also Supplementary Table 4.

The removal of glycans at position N160 in the V1V2 region generated a glycan hole that led to a significant difference in the number of glycan atoms per residue on the Env trimer between WT and N160K (Fig. 5a bottom). This further resulted in a substantial difference in epitope-glycan coverage between WT and N160K Env trimer for antibodies approaching this region (e.g., VRC26.25, PGT145, PG9, VRC38.01) (Fig. 5a top).

Interestingly, in spite of PGT145 and 2909 binding to the same V1V2 region, we observed far different glycan coverage from these two antibodies’ epitopes (Fig. 5a). The main reason for the discrepancy between these two epitopes arises from the definition of glycan coverage—the number of glycan atoms per surface area of the epitope. In other words, the epitopes of two different antibodies targeting the same region with similar number of glycan atoms may have significantly different surface areas corresponding to their AFADs relative to the mean. Antibody 2909 has a much larger protein epitope surface than PGT145 (Fig. 5b and Supplementary Table 4), while there was only a subtle discrepancy in the average number of glycan atoms between WT and N160K epitopes of PGT145 (392.79) and 2909 (323.92) (Supplementary Table 4). Consequentially, this leads to the 2909 epitope having less than half the glycan coverage compared to the PGT145 epitope.

## Discussion

In this study, we systematically analyzed the AFADs of all antibody–antigen complexes available in the Protein Data Bank^7^, and showed that the longest AFADs were from antibodies targeting the HIV-1 Env. Specifically, these HIV-1 antibodies with long AFADs target the V1V2, glycan-V3, and silent face sites of HIV-1 Env, all of which are densely glycosylated. We found that only broadly neutralizing HIV-1 antibodies show the extended AFADs relative to the mean, while none of the other antibodies targeting viral antigens, such as Ebola virus glycoprotein, influenza A virus hemagglutinin, Dengue/Zika envelope, or SARS-CoV-2 spike, had significantly extended AFADs relative to mean. HIV-1 Env has the most dense glycan coverage (Supplementary Fig. 7), especially in certain regions (V1V2, glycan-V3, and silent face sites), which explains why the exceptionally long AFADs were only observed for HIV-1 antibodies. The significant correlation between AFAD and glycan density demonstrated the dependency of these two properties.

We have used the distance between conserved Fv cysteines and antigens to define AFAD. A main caveat of this method is that an exceptionally short AFAD relative to the mean can result from an antigen interacting with the side of the antibody (Supplementary Fig. 1). Also, we built Man-9 models on each of the *N*-linked glycosylation sequons for MD simulations and this might not precisely capture the actual glycan coverage^19^. Finally, we have shown one example with 2909, an antibody targeting a strain-specific glycan hole, which has a shorter AFAD relative to the mean at the densely glycosylated V1V2 site. The removal of three N160 glycans from the apex of the trimer mainly led to a considerably shorter AFAD relative to the mean. Shorter AFADs relative to the mean were also observed for antibodies targeting glycan holes in the V3 region, such as antibodies R56^20^ (−4.38 Å) and R20^20^ (3.51 Å), which were elicited from immunization of rabbits with gp120 of clade B strain JR-FL, supporting the observation that antibodies binding to glycan holes generally do not have extended AFADs relative to mean.

Of note, there was only a low correlation observed between epitope-glycan content and glycan coverage (Supplementary Fig. 4d), and the correlation of AFAD to epitope-glycan content (Supplementary Fig. 3d) was also much lower than that to glycan coverage (Fig. 3c). Glycan coverage quantifies the density of glycan shield that an antibody needs to overcome (e.g., through extended AFAD), but antibodies, such as fusion peptide-targeting VRC34.01 and PGT151, can certainly have short AFADs and have substantial contact with glycans when binding, given that the glycan shield is not too dense (i.e., low glycan coverage).

Understanding the relationship between antibody properties and antigen recognition is critical for the design of B cell-based vaccines. This is particularly important for HIV-1 vaccine development as there are many obstacles, such as sequence diversity, conformational flexibility, and dense glycosylation of the HIV-1 Env trimer that have impeded the development of an effective HIV-1 vaccine^21^. Previously, we have analyzed several antibody properties, such as CDR H3 length, number of antibody protruding loops, level of somatic hypermutation, number of independent epitope segments and epitope-glycan components, and have found that epitope protein surface correlated with neutralization breadth and epitope glycan component inversely correlated with neutralization breadth^8^. Continuing research of antibody properties, quantification of AFAD at the molecular level in this work provides insight into antibody-guided vaccine design. The remarkable correlation between AFAD relative to the mean and glycan density suggests that it might be reasonable to focus on vaccine response toward glycan-depleted regions^22–24^, as antibodies targeting glycan-dense region often requires longer AFAD, which may be difficult to elicit. Alternatively, we note that the three antibodies with the largest AFADs targeting the most glycan-dense sites were some of the most potent broadly neutralizing antibodies thus far identified; despite their being difficult to elicit, the field may want to investigate vaccine regimes that can coax the immune system into eliciting antibodies with long AFADs capable of effectively targeting glycan-dense regions.

## Methods

### Antibody–antigen complexes

More than 4500 PDBs of antibody–antigen complexes with high resolution (<3 Å) were obtained from SAbDab^25^ on November 5, 2020. To remove redundancy of ~4500 PDBs, CD-HIT^26,27^ was used to cluster the antibody–antigen complexes. A single input file that includes a full antibody set of heavy and light chain concatenated sequences was entered. CD-HIT clustered the entire set and yielded 1879 non-redundant antigen–antibody complexes using 95% sequence identity and 99% of alignment coverage for the longer sequence, while excluded gaps when calculating identity. The computational time spent ~1 s with one core of CPU, 2.4 GHz (Intel Xeon E5-2680v4). For HIV-1 antigen–antibody complexes specifically, we used the 20 complexes as described in Chuang et al.^8^, plus the addition of SF12 (PDB:6OKP)^28^, CAP256-VRC26.25 (PDB:6VTT)^12^, and 1-18 (PDB:6UDJ)^29^ (Supplementary Table 5).

### Calculation of AFAD

We measured the distances between any heavy atom on the antigen and any heavy atom on the four conserved cysteines—C22 and C92 on the heavy chain, and C23 and C88 on the light chain—of the antibody four conserved cysteines—C22 (${{{{{{\rm{D}}}}}}}_{1}$) and C92 (${{{{{{\rm{D}}}}}}}_{2}$) on the heavy chain, and C23 (${{{{{{\rm{D}}}}}}}_{3}$) and C88 (${{{{{{\rm{D}}}}}}}_{4}$) on the light chain—of the antibody.

1 $${{{{{{\rm{D}}}}}}}_{i}=\sqrt{{({x}_{i{{{{{\rm{a}}}}}}}-{x}_{i{{{{{\rm{b}}}}}}})}^{2}+{({y}_{i{{{{{\rm{a}}}}}}}-{y}_{i{{{{{\rm{b}}}}}}})}^{2}+{({z}_{i{{{{{\rm{a}}}}}}}-{z}_{i{{{{{\rm{b}}}}}}})}^{2}},$$

where $({x}_{i{{{{{\rm{a}}}}}}},{y}_{i{{{{{\rm{a}}}}}}},{z}_{i{{{{{\rm{a}}}}}}})$ are the coordinates of the closest atom in antigen, $({x}_{i{{{{{\rm{b}}}}}}},{y}_{i{{{{{\rm{b}}}}}}},{z}_{i{{{{{\rm{b}}}}}}})$ are the coordinates of the *i*-th conserved cysteine in variable domain of antibody. Then, AFAD was defined as the minimum value of four distances.

2 $${{\mbox{AFAD}}}={\min }{{\mbox{(}}}{{{\mbox{D}}}}_{1}{{\mbox{,}}}{{{\mbox{D}}}}_{2}{{\mbox{,}}}{{{\mbox{D}}}}_{3}{{\mbox{,}}}{{{\mbox{D}}}}_{4}{{\mbox{)}}},$$

### Glycan coverage analysis

We used an in-house program, GLYCO, to quantify the glycan shield of Env trimer. The method counts the number of glycan atoms of glycosylated protein structures within a certain radial distance cutoff (e.g., 26 Å) from surface protein residues, while excluding ones that are blocked by protein region. The counted glycan atom numbers are averaged over 500 frames (one frame per one ns) of 500 nanosecond (ns) MD simulations. The cutoff was determined by analyzing the longest length of the glycan in Env trimer averaged over 500 ns of MD simulations. The surface residues were evaluated by their solvent accessible surface area (SASA) by FreeSASA^30^ with 5 Å of probe radius. The residues with larger than 30 Å^2^ of their surface area were selected as surface residues.

Epitope-glycan coverage was calculated by counting the number of glycan atoms of epitope residues of the glycosylated protein and dividing by surface area of the epitopes. The number of glycan atoms per epitope residue was analyzed with the same method and same parameters described above. The summation of the epitope residues’ buried SASA was used as surface area of the epitope to normalize the average number of glycan atoms (see more details in “Epitope residue, epitope total/protein surface area, and paratope surface area determination” section).

### Molecular dynamics simulations

Molecular dynamics (MD) simulations were carried out to estimate the conformational flexibility of HIV-1 Env protein under physiological conditions. Initial atomistic model of HIV-1 Env protein was generated using YASARA software’s (http://www.yasara.org/) homology modeling plugin with default option. HIV-1 Env was modeled using clade A BG505 wild-type sequence as target and 5 trimeric Env structures as templates (PDBs: 5FYJ, 5FYK, 5FYL, 6CK9, 6MUF). Lassa virus GPC using one template (PDB ID: 5VK2), SARS-CoV-2 spike using one template (PDB ID: 6VXX), influenza virus HA using one template (PDB ID: 6CF7), RSV F using one template (PDB ID: 4MMS), and Zika virus E protein using one template (PDB ID: 5JHM). All potential *N*-linked glycosylation sites were modeled with Man-9 glycans (Figs. 3 and 5) and Man-5 glycans (Supplementary Fig. 7) using an in-house program Glycosylator^31^. Subsequently, the modeled trimeric Env structure was solvated in a 15 Å padding water box and neutralized by the addition of NaCl at a concentration of 150 mM. The final systems were composed of 555,778 (HIV-1 Env), 224,785 (Lassa virus GPC), 759,908 (SARS-CoV-2 spike), 265,711 (influenza virus HA), 258,677 (RSV F), and 150,576 (Zika virus E) atoms. The MD was performed using NAMD2.13 engine^32^, with CHARMM36 force field^33,34^. TIP3P water parameterization was utilized to describe the water molecules^35^. The periodic electrostatic interactions were computed using particle-mesh Ewald (PME) summation^36^ with a grid spacing smaller than 1 Å. Constant temperature was imposed by using Langevin dynamics with a damping coefficient of 5.0 ps. The system was first minimized by 20,000 conjugate gradient steps and then equilibrated by using a linear temperature gradient, which heated up the system from 100 to 310 K in 2 ns plus additional 10 ns. The length of all bonds involving hydrogen atoms was constrained with the RATTLE algorithm^37^, thus allowing a time step of 2 fs. Unrestrained molecular dynamics were additionally performed up to 500 ns and used in the analysis of glycan conformations.

### Expression and purification of CAP256 Env trimer

An N160K variant of the CAP256.wk34.c80 SOSIP.RnS2 trimer^12^ was generated using site-directed mutagenesis (GeneImmune Biotechnology, Rockville, MD) and soluble Env trimers were produced by transient transfection in FreeStyle 293-F cells^38^. The Freestyle 293-F cells were transfected with 600 μg of Env trimer plasmid along with 150 μg of human furin plasmid DNA per liter of cells using 293Fectin (Thermo Scientific) according to the manufacturer’s directions. Transfected cells were incubated in a shaker at 120 rpm, 37 °C, and 9% CO_2_. The following day, 80 ml HyClone SFM4HEK293 medium and 80 ml FreeStyle 293 Expression Medium were added to each liter of cells. Culture supernatants were harvested by centrifugation 6 days following transfection and filtered. CAP256 Env trimers were purified using either 2G12 affinity chromatography, with low pH elution, followed by gel filtration on a Superdex 200 column in PBS^12^ or by binding to a cleavable version of the CD4-binding site antibody, 3BNC117^39^ to facilitate purification. In this case, 250 ml of cell supernatant, harvested from transiently transfected cells expressing 3BNC117 antibody with a HRV3C cleavage site in the heavy-chain hinge region, as described below, was added to the clarified supernatant prior to loading onto a protein A column. Following capture of the Env trimer-3BNC117 complex onto protein A resin, HRV3C protease was added to generate an Env trimer-3BNC117 Fab complex that was eluted from the column in PBS buffer. Env trimers and complexes were purified by gel filtration using a Superdex S-200 column equilibrated in PBS. Antigenicity and structural integrity of purified trimers were confirmed by biolayer interferometry binding and negative-stain electron microscopy analyses.

### Expression and purification of antibodies

Variable regions for 2909^9^, 3BNC117^39^, PGT145^16,17^, and 2G12^40^ were codon-optimized (Supplementary Table 6), synthesized, and subcloned (Gene Universal Inc, Newark, DE) into pVRC8400 (CMV/R) expression vectors containing human IgG constant regions. A heavy-chain plasmid containing an HRV3C cleavage site in the hinge region was used for Fab production. Corresponding heavy and light chain plasmids were co-transfected in Expi293F cells using Turbo293 transfection reagent^41^. Briefly, 0.75 ml of Turbo293 transfection reagent (Speed BioSystems) were added to 12.5 ml Opti-MEM medium (Life Technology) and incubated for 5 min at room temperature. Meanwhile, 125 μg of heavy chain and 125 μg of light chain plasmid DNA were added to 12.5 ml of Opti-MEM medium in another tube. The Opti-MEM medium containing Turbo293 was then added to plasmid DNAs, incubated for 15 min at room temperature, and added to 200 ml of Expi293 cells (Life Technology) at 2.5 million cells/ml. The transfected cells were cultured in shaker incubator at 120 rpm, 37 °C, 9% CO_2_. Culture supernatant was collected 5 days after transfection and loaded onto a protein columns. After washing the columns with PBS, IgG was eluted using a low pH buffer and immediately neutralized with 10% 1 M Tris-Cl, pH 8.0. To produce 2909 Fab, purified IgG was cleaved by HRV3C protease and the digest was passed over a protein A column to separate the Fab fragments. The Fab fragments were purified further by size-exclusion chromatography using a Superdex 200 column in a buffer containing 5 mM HEPES, pH 7.5, and 150 mM NaCl.

### Biolayer interferometry assay

Binding of CAP256 Env trimers to HIV-1 antibodies was assessed using an Octet HTX instrument, as previously described^38^. Briefly, all assays were carried out with agitation set to 1000 rpm in PBS using solid black 96-well plates (Geiger Bio-One) at 30 °C. For the assessment, IgG antibodies were immobilized onto an AHC capture tip for 180 s at 50 µg/ml in PBS. Biosensor tips were then equilibrated for 300 s prior assessment of binding to the HIV-1 trimer molecules in solution (at 50 µg/ml). Association was allowed to proceed for 180 s followed by dissociation for 300 s. Data analysis was performed in ForteBio Data Analysis 11.0.

### Epitope residue, epitope total/protein surface area, and paratope surface area determination

FreeSASA^30^ was used to determine the epitope residues, epitope total surface area, epitope protein surface area, and paratope surface area. The epitope residues were defined by the residues having non-zero value of their SASA between antibody–antigen complex structure and antigen structure. Summation of the SASA of epitope residues is epitope total surface area and summation of only protein epitope residues is epitope protein surface area. The paratope surface area was calculated in the same manner as epitope surface area but between antibody–antigen complex structure and antibody structure.

### Epitope-glycan percentage

The epitope-glycan percentage was determined by the dividing the SASA of glycan epitope residues by the SASA of the total epitope residues.

### Epitope conservation

Residue conservation (percentage of the most prevalent residue type) was calculated based on the filtered web alignment of the year 2018 from the Los Alamos HIV sequence database (http://www.hiv.lanl.gov/). Alignment gap was treated as an additional residue type. Epitope conservation was determined based on average of the residue conservation among the epitope residues.

### CDR H3, CDR L3 length, and somatic hypermutation (SHM)

Antibody CDR H3 and L3 length were identified with IMGT numbering scheme by ANARCI^42^. The sequences between the second conserved C and WGXG (where X is any residue) were counted as CDR H3 length. CDR L3 length was defined by the sequences between the conserved C and FGXG (where X is any residue). The SHM was examined with the IGBLAST webserver (https://www.ncbi.nlm.nih.gov/igblast).

### Virus neutralization

Single-round-of-replication Env pseudoviruses were prepared, titers were determined, and the pseudoviruses were used to infect TZMbl target in an optimized and qualified automated 384-well format^43^. Briefly, antibodies were serially diluted, a constant amount of pseudovirus added, and plates incubated for 60 min; followed by addition of TZMbl cells which express luciferase upon viral infection. The plates were incubated for 48 h and then lysed, and luciferase activity measured. Percent neutralization was determined by the equation: (virus only) − (virus+antibody)/(virus only) multiplied by 100. Data are expressed as the antibody concentration required to achieve 50% neutralization (IC50) and calculated using a dose-response curve fit with a 5-parameter nonlinear function (Supplementary Data 1). We used a previously described panel^8,44–46^ of 208 geographically and genetically diverse Env pseudoviruses representing the major subtypes and circulating recombinant forms.

### Cryo-EM data collection and processing

The CAP256.wk34.c80 SOSIP.RnS2 trimer with the N160K mutation complexed with 3BNC117 was incubated with molar excess of 2909 Fab fragments. A volume of 2.1 μl of the complex at 2 mg/ml concentration was deposited on a C-flat 1.2/1.3 grid (protochip.com) then vitrified using an FEI Vitrobot Mark IV with a wait time of 30 s, blot time of 3 s, and blot force of 1. Data collection was performed on a Titan Krios electron microscope with Leginon^47^ using a Gatan K2 Summit direct detection device. Exposures were collected in movie mode for a 10 s with the total dose of 66.88 e^−^/Å^2^ fractionated over 50 raw frames. Images were pre-processed using Appion^48,49^; individual frames alignment and dose-weighted were executed using MotionCor2^50^. CTFFind4^51,52^ was used to estimate the CTF and DoG Picker^48,49^ was used to pick particles. RELION^53^ was used for particle extraction and the particle stack was transferred to cryoSPARC. cryoSPARC 2.15^54^ was used for 2D classifications, ab initio 3D reconstruction, homogeneous refinement, and nonuniform 3D refinement. Initial 3D reconstruction and final refinements were performed using C1 symmetry, confirming three 3BNC117 Fab molecules and one 2909 Fab per trimer. The final resolution of the C1 nonuniform refinement was 3.91 Å.

Coordinates from PDB IDs 6VRW^12^ and 3PIQ^14^ were used for initial fit to the reconstructed map. This was followed by simulated annealing and real-space refinement in Phenix^55^ with the sharpened map from cryoSPARC and with a density modified map from Phenix Resolve^56^ and then iteratively processed with manual fitting of the coordinates in Coot^57^. Geometry and map fitting were evaluated throughout the process using Molprobity^58^ and EMRinger^59^. PyMOL (www.pymol.org) and ChimeraX^60^ were used to generate figures.

### Reporting summary

Further information on research design is available in the Nature Research Reporting Summary linked to this article.

## Supplementary information

Supplementary Information

Description of Additional Supplementary Files

Supplementary Data 1

Reporting summary

## Data Availability

Cryo-EM maps and fitted coordinates have been deposited with EMDB entry ID EMD-23589 and PDB entry ID 7LY9, respectively. More than 4500 antibody dataset is available at SabDab. Source data are provided with this paper.

## Source data

Source Data

## Code availability

The in-house program GLYCO is available at https://github.com/myungjinlee/GLYCO.
