# Supplementary material for: Extended antibody-framework-to-antigen distance observed exclusively with broad HIV-1-neutralizing antibodies recognizing glycan-dense surfaces
Source: Nat Commun. 2021 Nov 9;12:6470. doi: 10.1038/s41467-021-26579-z (PMC8578620; doi:10.1038/s41467-021-26579-z)
Supplement: Supplementary file 1 — Supplementary Information [file 41467_2021_26579_MOESM1_ESM.pdf]

Supplementary Information

Extended Antibody-Framework-to-Antigen Distance Observed Exclusively with Broad HIV-1-Neutralizing Antibodies Recognizing Glycan-Dense Surfaces

Myungjin Lee[1,*], Anita Changela[1,*], Jason Gorman[1,*], Reda Rawi[1], Tatsiana Bylund[1], Cara W. Chao[1], Bob C. Lin[1], Mark K. Louder[1], Adam S. Olia[1], Baoshan Zhang[1], Nicole A. Doria-Rose[1], Susan Zolla-Pazner[2], Lawrence Shapiro[1,3], Gwo-Yu Chuang[1,†], and Peter D. Kwong[1,3†]

[1] Vaccine Research Center, NIAID, National Institutes of Health, Bethesda, MD 20892, USA.

[2] Department of Medicine and Department of Microbiology, Icahn School of Medicine at Mount Sinai, New York, NY 10029, USA.

[3] Department of Biochemistry and Molecular Biophysics, Columbia University, New York, NY 10032, USA.

[*] These authors contributed equally.
† Corresponding authors. E-mail: (GYC) gwo-yu.chuang@nih.gov; (PDK) pdkwong@nih.gov

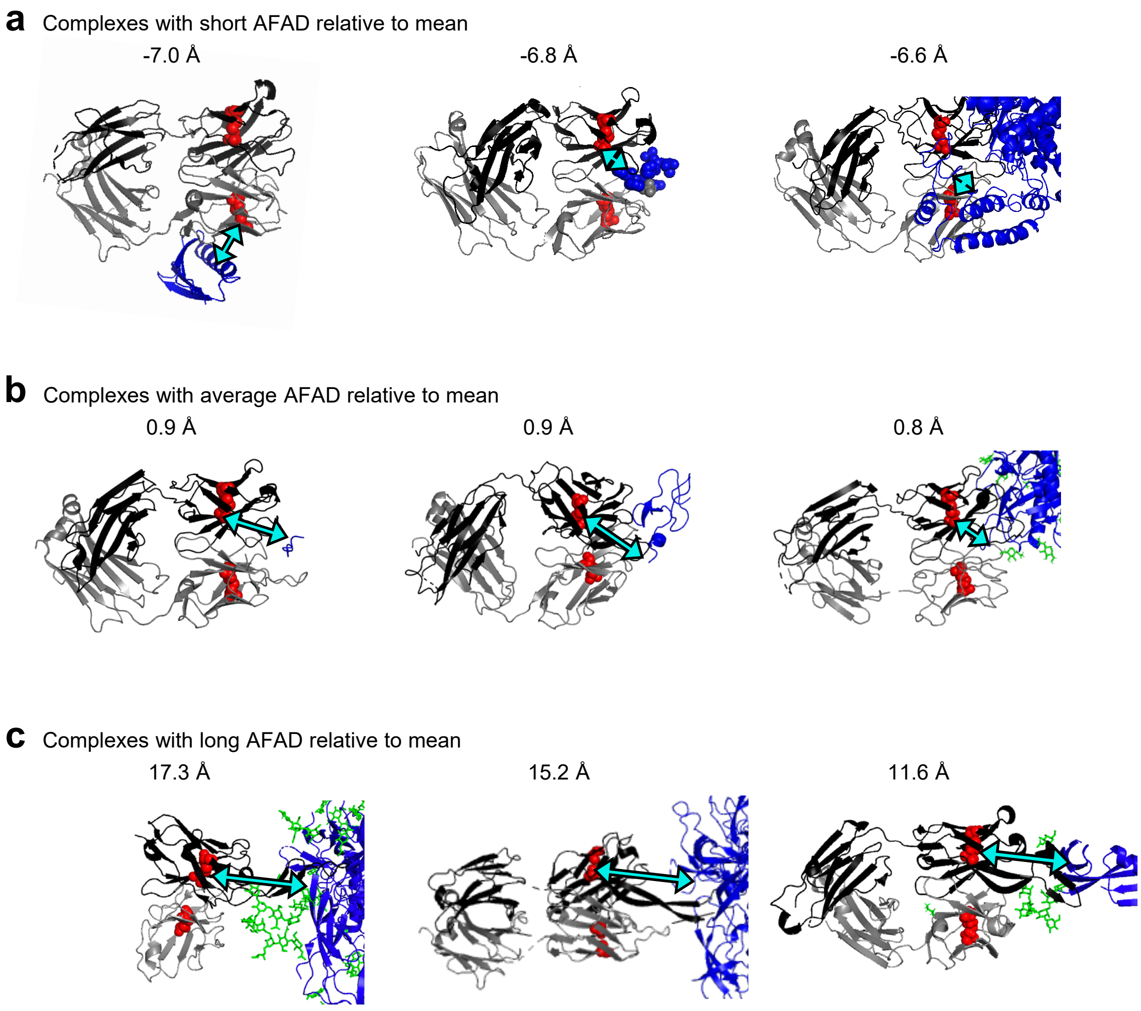

Supplementary Figure 1 | Select examples of complexes with short, average, or long AFAD relative to mean. Heavy chains are colored in black, light chains are in grey, the conserved cysteines are in red, antigen proteins are in blue, glycans are in green, and AFADs are shown as cyan double headed arrows.

(a) Select complexes with short AFAD relative to mean, including complex of *Peptostreptococcus magnus* protein L and human antibody Fab 2A2 (PDB: 1HEZ) (left), Fab/epitope complex of human chimeric monoclonal antibody h4E6 targeting a phosphorylated tau epitope (PDB: 6DC9) (middle), and Taq DNA polymerase in complex with Fab TP7 (PDB: 1BGX) (right). (b) Select complexes with average AFAD relative to mean, including 580 germline antibody bound to circumsporozoite protein NANP 5-mer (PDB: 6AZM) (left), structure of the human 4-1BB and Urelumab Fab complex (PDB:6MHR) (middle), and antibody 1B2530 in complex with HIV-1 clade A/E 93TH057 gp120 (PDB: 4YFL) (right). (c) Select complexes with long AFAD relative to mean, including HIV-1 Env Trimer complex with antibody VRC26.25 Fab (PDB: 6VTT) (left), HIV-1 Env Trimer complex with PGT145 (PDB: 5U1F) (middle), and HIV-1 Env Trimer complex with antibody PG9 (PDB: 3U2S) (right). Cyan arrows denote the residue pairs from which the AFAD were calculated.

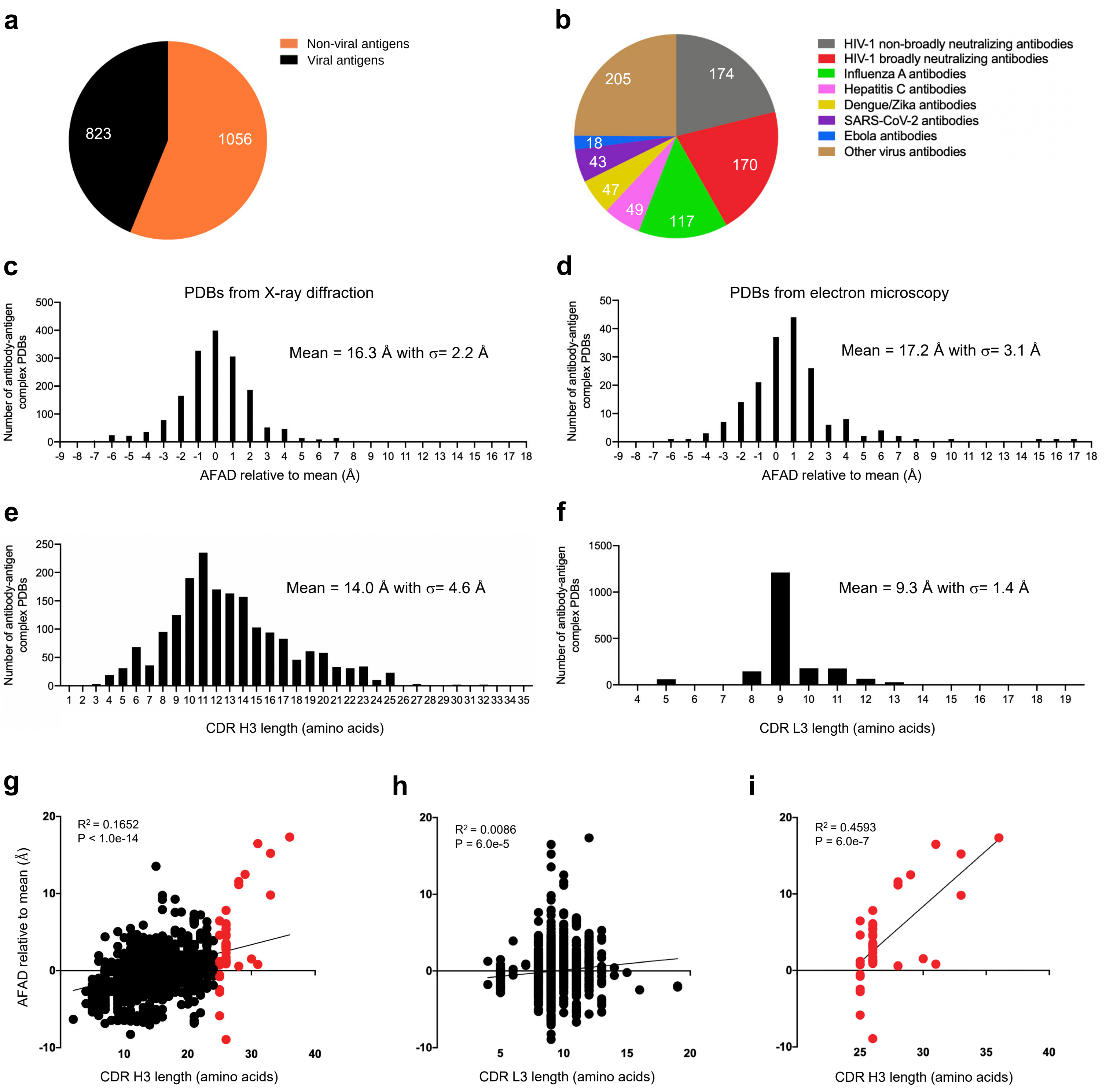

Supplementary Figure 2 | Composition of antibody-antigen complex PDBs, and histograms and correlations of AFAD relative to mean with antigen properties.

(a) Overall composition of antibody-antigen complexes PDB collection. (b) The distribution of viral antigens. (c) Histograms of AFAD of antibodies from X-ray diffraction structures. n=1695 antibody-antigen complex PDBs. Data are presented as mean values 16.3 +/- 2.2 Å SD. (d) Histograms of AFAD of n=181 antibodies from electron microscopy (EM) structures. Data are presented as mean values 17.2 +/- 3.1 Å SD. (e) Histograms of CDR H3 length of all n=1879 antibodies. Data are presented as mean values 14.0 +/- 4.6 Å SD. (f) Histograms of CDR L3 length of all n=1879 antibodies. Data are presented as mean values 9.3 +/- 1.4 Å SD. (g) Correlation between AFAD relative to mean for all n=1879 antibodies and CDR H3 length by simple linear regression. The antibodies with long CDR H3 length (> 24) are colored in red. (h) Correlation between AFAD relative to mean for all n=1879 antibodies and CDR L3 length by simple linear regression. (i) Correlation between AFAD relative to mean for n=43 antibodies with CDR H3 length longer than 24 amino acids by simple linear regression.

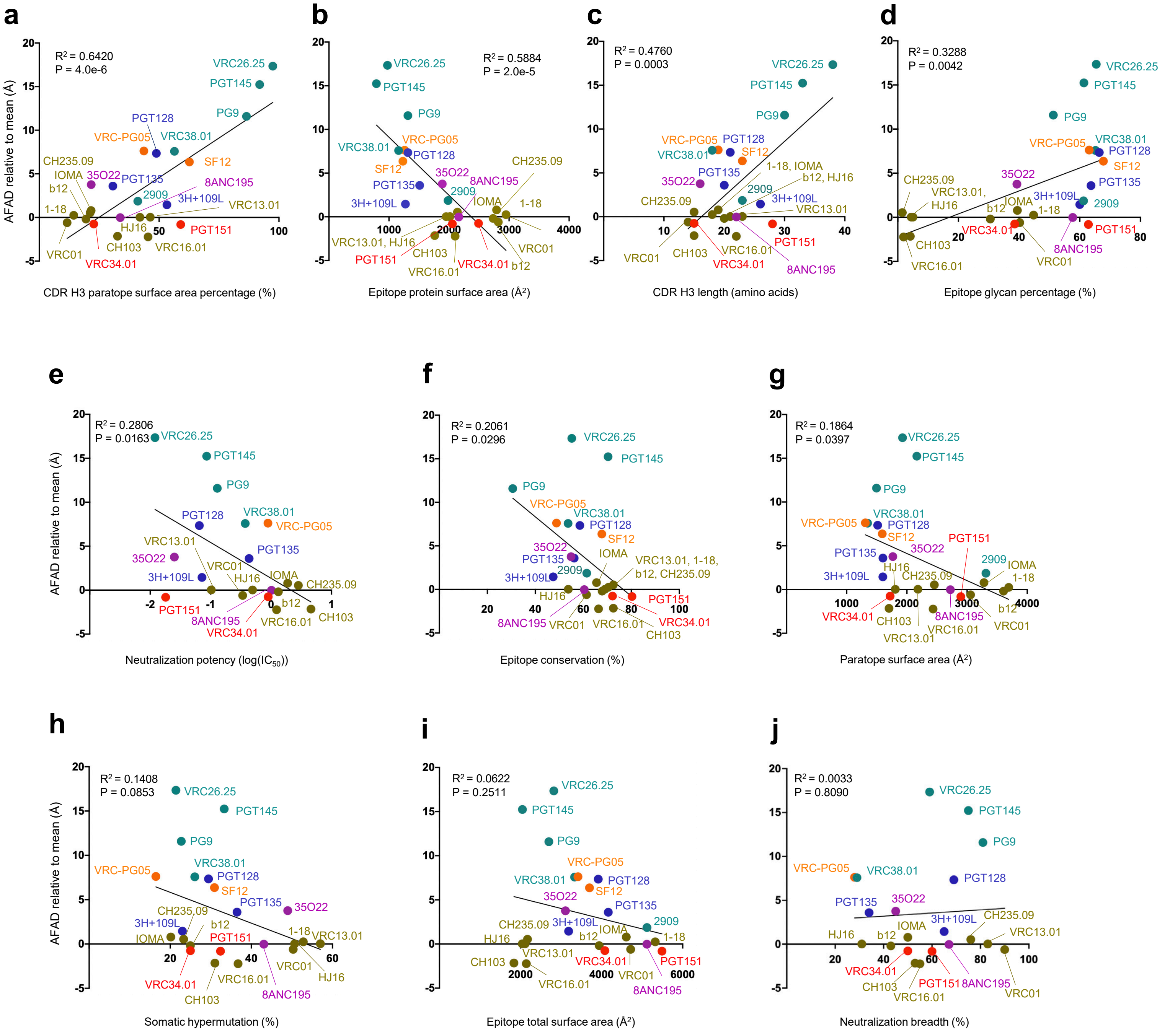

Supplementary Figure 3 | Correlation of AFAD relative to mean and antigen properties by simple linear regressions. See Supplementary Table 5 for PDB code of antibodies. Antibodies are colored by epitope categories, as shown in Fig. 2.

(a) AFAD relative to mean versus CDR H3 paratope surface area percentage of n=23 antibodies. 2G12 is excluded.
(b) AFAD relative to mean versus epitope protein surface area of n=23 antibodies. 2G12 is excluded.
(c) AFAD relative to mean versus CDR H3 length of n=23 antibodies. 2G12 is excluded.
(d) AFAD relative to mean versus epitope glycan percentage of n=23 antibodies. 2G12 is excluded.
(e) AFAD relative to mean versus neutralization potency of n=20 antibodies (geometric mean from a 208-virus neutralization panel), unit of IC_{50}: µg/ml. 2G12, 2909, SF12, and 1-18 are excluded.
(f) AFAD relative to mean versus protein epitope conservation of n=23 antibodies. 2G12 is excluded.
(g) AFAD relative to mean versus paratope surface area of n=23 antibodies. 2G12 is excluded.
(h) AFAD relative to mean vs somatic hypermutation (SHM) of n=22 antibodies. 2G12 and 2909 are excluded.
(i) AFAD relative to mean versus epitope total surface area of n=23 antibodies. 2G12 is excluded.
(j) AFAD relative to mean versus neutralization breadth of n=20 antibodies. 2G12, 2909, SF12, and 1-18 are excluded.

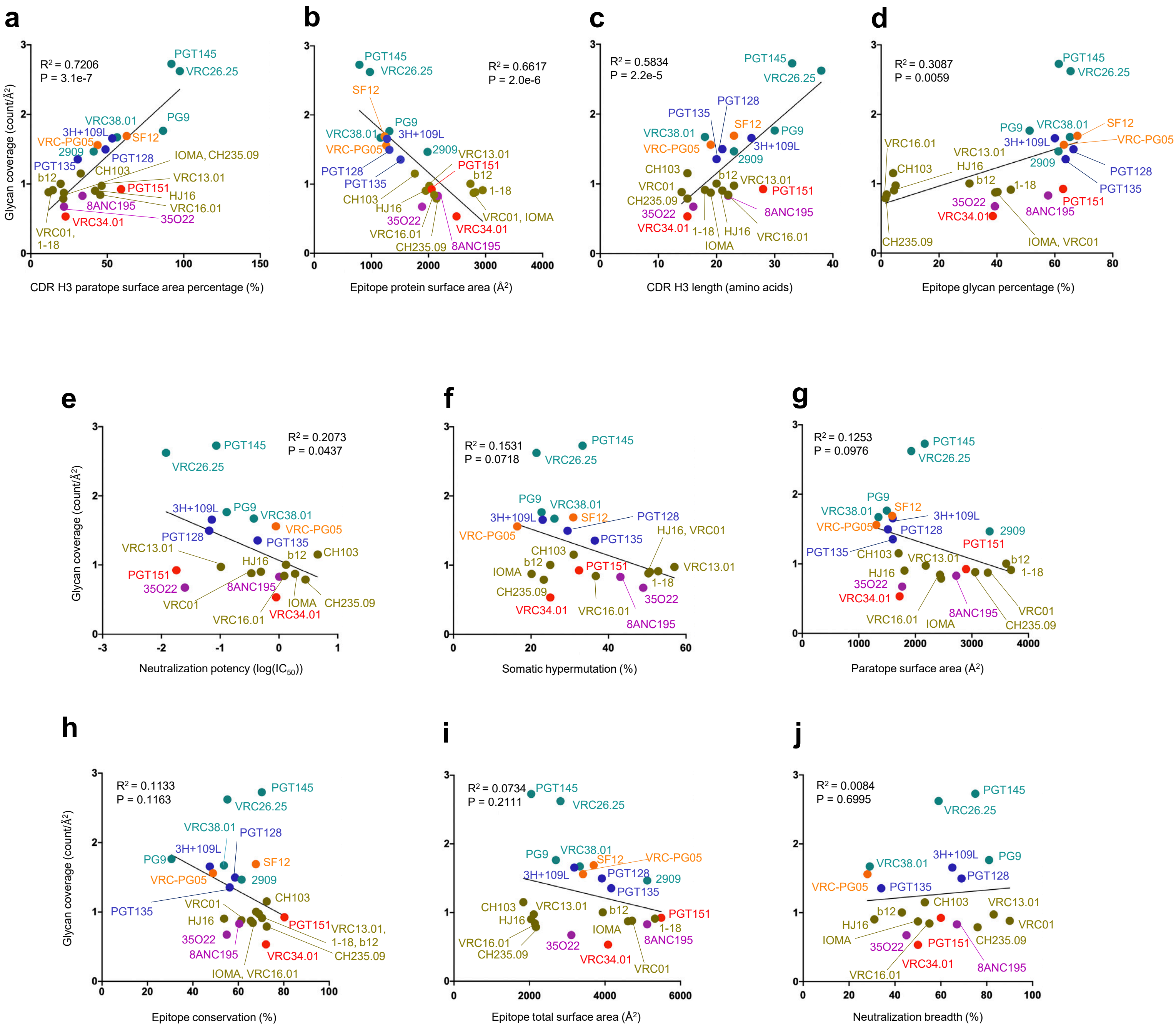

Supplementary Figure 4 | Correlation of glycan coverage and antigen properties by simple linear regressions.
See Supplementary Table 5 for PDB code of antibodies. Antibodies are colored by epitope categories, as shown in Fig. 2.

(a) Glycan coverage versus CDR H3 paratope surface area percentage of n=23 antibodies. 2G12 is excluded.
(b) Glycan coverage versus epitope protein surface area of n=23 antibodies. 2G12 is excluded.
(c) Glycan coverage versus CDR H3 length of n=23 antibodies. 2G12 is excluded.
(d) Glycan coverage versus protein epitope glycan percentage of n=23 antibodies. 2G12 is excluded.
(e) Glycan coverage versus neutralization potency of n=20 antibodies (geometric mean from a 208-virus neutralization panel), unit of IC50: μg/ml. 2G12, 2909, SF12, and 1-18 are excluded.
(f) Glycan coverage vs somatic hypermutation (SHM) of n=22 antibodies. 2G12 and 2909 are excluded.
(g) Glycan coverage versus paratope surface area of n=23 antibodies. 2G12 is excluded.
(h) Glycan coverage versus epitope conservation of n=23 antibodies. 2G12 is excluded.
(i) Glycan coverage versus epitope total surface area of n=23 antibodies. 2G12 is excluded.
(j) Glycan coverage versus neutralization breadth of n=20 antibodies. 2G12, 2909, SF12, and 1-18 are excluded.

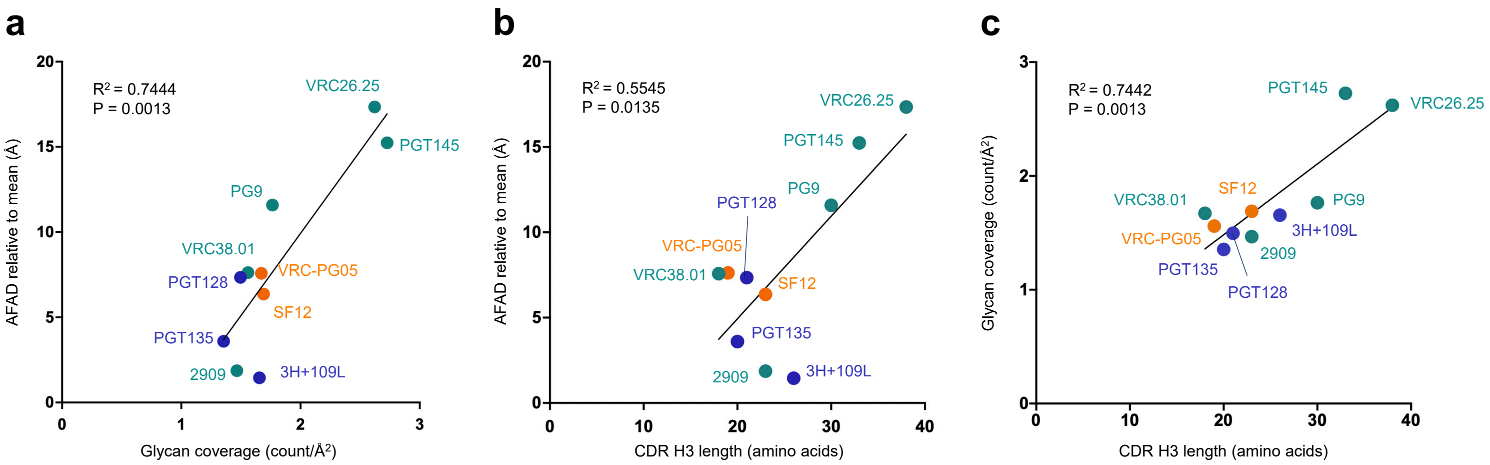

Supplementary Figure 5 | Correlation between AFAD relative to mean or glycan coverage of antibodies with glycan coverage higher than average and antigen properties by simple linear regressions.

See Supplementary Table 5 for PDB code of antibodies. Antibodies are colored by epitope categories, as shown in Fig. 2.

(a) AFAD relative to mean versus glycan coverage of n=10 antibodies that have higher than average of glycan coverage.

(b) AFAD relative to mean versus CDR H3 length of n=10 antibodies that have higher than average of glycan coverage.

(c) Glycan coverage versus CDR H3 length of n=10 antibodies that have higher than average of glycan coverage.

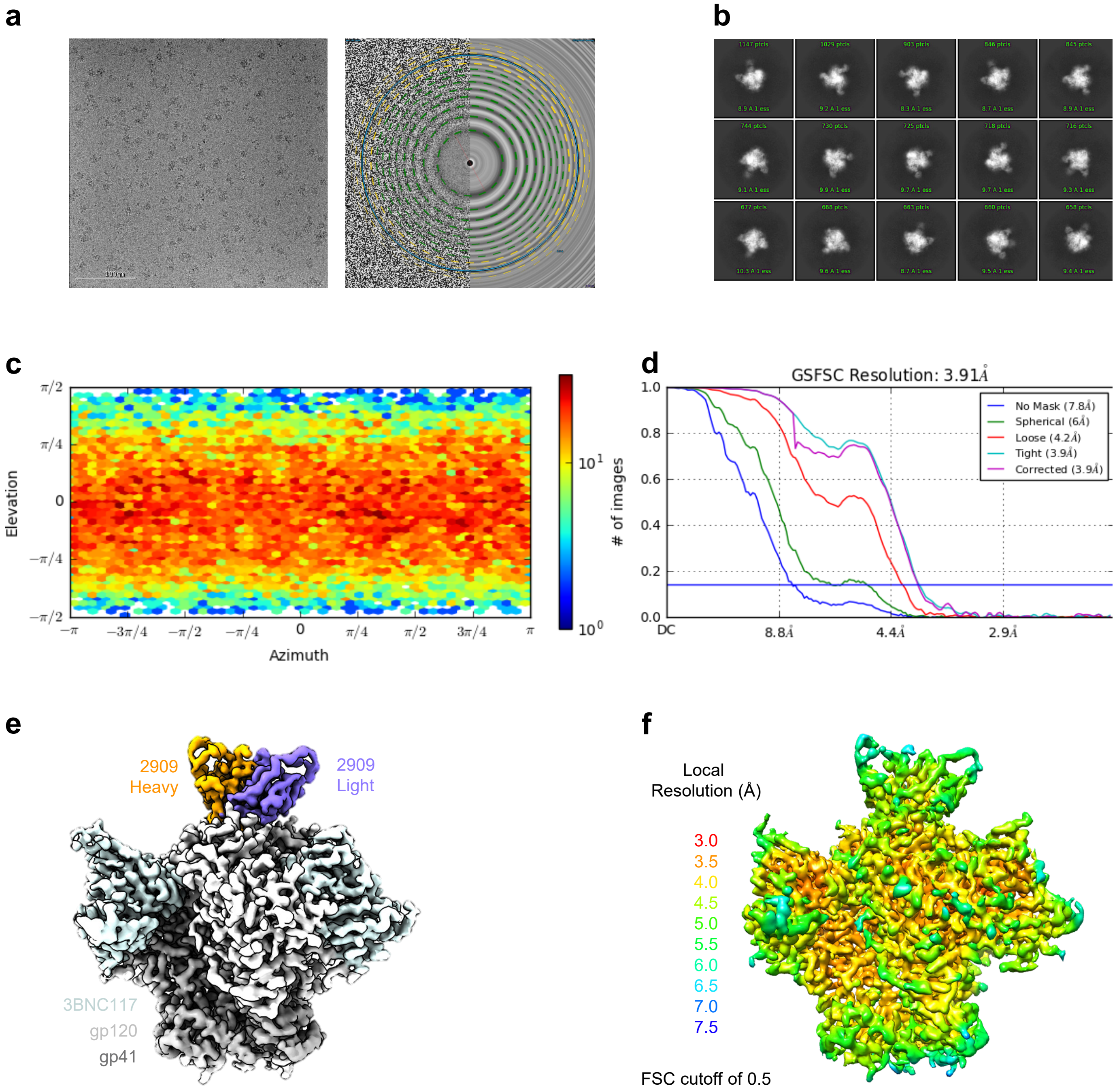

Supplementary Figure 6 | Cryo-EM Details of 2909 Fab in complex with 3BNC117 and CAP256.wk34.c80 SOSIP.RnS2 N160K HIV-1 Env trimer.

(a) Representative micrograph and CTF of the micrograph are shown. A 100 nm scale bar is shown in white in the lower left corner of the micrograph.1,428 total micrographs were imaged from one grid for the structure.

(b) Representative 2D class averages are shown.

(c) The orientations of all particles used in the final refinement are shown as a heatmap.

(d) The gold-standard fourier shell correlation resulted in a resolution of 3.91 Å using non-uniform refinement with C1 symmetry.

(e) Density for the complex structure is shown with the heavy chain in orange and light chain in slate-blue.

(f) The local resolution of the full map is shown generated through cryoSPARC using an FSC cutoff of 0.5.

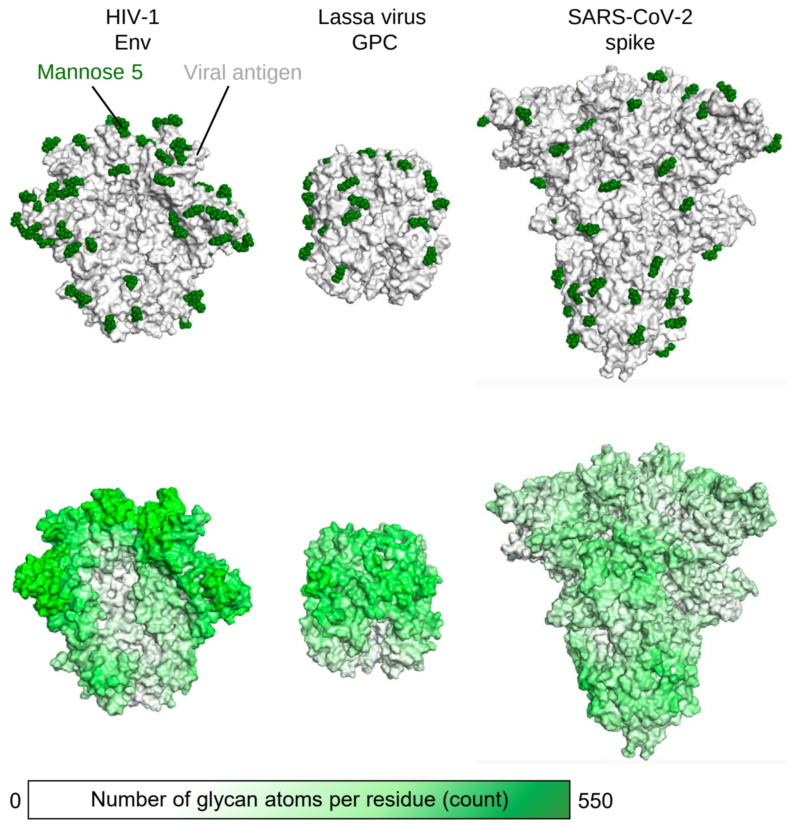

Supplementary Figure 7 | Positions of *N*-linked glycosylation (displayed as modeled mannose-5 in green) glycans on viral antigens HIV-1 envelope trimer (Env), Lassa virus glycoprotein (GPC), SARS-CoV-2 spike, influenza virus hemagglutinin (HA) H1 Solomon Islands 06, respiratory syncytial virus fusion glycoprotein (RSV F), Zika virus E protein (top row) versus GLYCO-calculated glycan coverage (bottom row).

Supplementary Table 1 | Extended AFADs relative to mean (top 50 among 1879).

Rank	PDB	Heavy chain	Light chain	Antigen chain	AFAD relative to mean	Category	Antibody
1	6vtt	H	L	F	17.34	HIV-1 broadly neutralizing antibody (bNAb)	VRC26.25
2	5v8l	J	N	A	15.23	HIV-1 bNAb	PGT145
3	6o41*	B	A	O	13.55	HIV-1 Ab	PGZL1
4	4dqo	H	L	C	12.50	HIV-1 bNAb	PG16
5	3u2s	H	L	G	11.58	HIV-1 bNAb	PG9
6	6e5p	H	L	A	7.93	HIV-1 bNAb	2G12
7	5esz	A	B	C	7.83	HIV-1 bNAb	CH04
8	6bf4	B	C	A	7.62	HIV-1 bNAb	VRC-PG05
9	5vgj	H	L	G	7.57	HIV-1 bNAb	VRC38.01
10	5aco	G	J	C	7.34	HIV-1 bNAb	PGT128
11	2ny5	H	L	G	6.94	HIV-1 NAb	17b
12	6cmg	C	B	A	6.47	Hendra Virus Ab	m102.3
13	6okp	Q	R	F	6.36	HIV-1 bNAb	SF12
14	5lcv	H	L	B	6.11	Zika and Dengue virus bNAb	EDE2A11
15	1tzi	B	A	V	5.93	Vascular endothelial growth factor a	YADS2
16	6x9r	H	L	D	5.83	HIV-1 Ab	RM20A3
17	6e3h	H	L	A	5.50	H3 influenza A hemagglutinin Ab	S9-3-37
18	4ut6	I	M	B	5.34	Dengue 2 virus bNAb	EDE2 B7
19	6vo0	H	L	A	5.28	HIV-1 NAb	43A2
20	6c9u	H	L	A	5.07	[KS3][AT3] didomain Ab	1B2
21	1ikf	H	L	C	5.04	Cyclosporin A Ab	IGG1-KAPPA R45-45-11
22	5bv7	C	B	A	4.99	Enzyme lecithin cholesterol acyltransferase Ab	27C3
23	5iq7	H	L	P	4.91	HIV-1 NAb	10E8-S74W
24	5e8e	B	A	H	4.91	Thrombin	Exosite 1-specific IgA Fab
25	4fp8	H	L	A	4.71	H3 influenza A hemagglutinin Ab	C05
26	5i6x	B	C	A	4.70	Human serotonin transporter Ab	8B6
27	6cmi	D	C	B	4.63	Hendra Virus Ab	m102.3
28	6xrt	H	L	E	4.56	HIV-1 Ab	VRC01.23
29	5dum	H	L	A	4.54	H5 Influenza A hemagglutinin Ab	65C6
30	5fyl	D	E	B	4.53	HIV-1 bNAb	35O22
31	5umn	E	F	A	4.50	H3 influenza A hemagglutinin Ab	C05 VPGSGW
32	6iuv	C	D	B	4.47	H5 influenza A hemagglutinin Ab	3C11
33	6p65	F	G	E	4.37	HIV Env 16055 NFL TD 2CC+	1C2
34	4m1c	E	F	B	4.32	Human Insulin Degrading Enzyme Ab	Amyloid-Beta (1-40)
35	3jbq	H	L	F	4.30	cGMP phosphodiesterase Ab	2E8
36	4iof	E	F	B	4.29	Insulin Degrading Enzyme Ab	Fab-bound IDE
37	6um7	J	K	A	4.21	HIV-1 NAb	DH270.mu1
38	5yhl	H	L	A	4.14	Prostaglandin E receptor EP4 Ab	Fab fragment
39	6um6	K	L	I	4.11	HIV-1 NAb	DH270.6
40	6u59	H	L	A	4.01	HIV-1 Ab	13B
41	6nb7	H	L	A	4.00	SARS-CoV Ab	S230
42	6mdt	D	E	B	3.98	HIV-1 bNAb	PGT124
43	4yhz	H	L	P	3.98	Histone methylation Ab	304M3-B
44	6bkd	H	L	E	3.90	Hepatitis C virus bNAb	AR3D
45	2b4c	H	L	G	3.88	HIV-1 Ab	X5
46	6q0e	H	L	A	3.87	H1 influenza A hemagglutinin Ab	Inferred precursor of the Human antibody lineage 652
47	3g04	B	A	C	3.84	Thyrotropin receptor Ab	M22
48	4yk4	C	B	A	3.83	H1 Influenza A hemagglutinin Ab	641 I-9
49	5zuf	E	D	C	3.82	Capsid protein VP2 Ab	R10
50	5u3o	H	L	A	3.82	HIV-1 Ab	DH511.2_K3

* 6o41 does not bind the antigen, membrane-proximal external region (MPER), but to G protein via the constant region.

Supplementary Table 2 | AFADs relative to mean for HIV-1 broadly neutralizing antibodies along with their recognition category and glycan coverage, as defined using various distance cutoff, proximal to each epitope.

Antibody	Recognition category	AFAD relative to mean (Å)	Glycan coverage proximal to each epitope for each cutoff from 5 to 35 Å (counts/Å^2)							
			5 Å	10 Å	15 Å	20 Å	25 Å	26 Å (selected cutoff)	30 Å	35 Å
VRC26.25	V1V2	17.34	0.10	0.38	1.16	1.92	2.61	2.78	3.24	3.82
PGT145		15.23	0.08	0.37	1.01	1.88	2.74	2.89	3.59	4.36
PG9		11.58	0.04	0.21	0.57	1.10	1.70	1.83	2.36	3.04
VRC38.01		7.57	0.06	0.24	0.60	1.07	1.58	1.68	2.09	2.58
2909	Hole-V1V2	1.86	0.06	0.33	0.73	1.12	1.45	1.51	1.74	2.01
PGT121-3H+109L	Glycan-V3	1.45	0.06	0.28	0.66	1.10	1.51	1.57	1.85	2.14
PGT128		7.34	0.07	0.25	0.54	0.93	1.33	1.39	1.71	2.09
PGT135		3.59	0.08	0.30	0.66	1.04	1.35	1.42	1.63	1.92
2G12		7.93	N/A	N/A	N/A	N/A	N/A	N/A	N/A	N/A
HJ16	CD4-binding site	0.02	0.05	0.21	0.44	0.69	0.92	0.97	1.13	1.36
IOMA		0.79	0.04	0.20	0.41	0.63	0.87	0.92	1.14	1.44
CH235.09		0.53	0.03	0.17	0.38	0.61	0.90	0.96	1.25	1.61
VRC01		-0.60	0.04	0.22	0.44	0.66	0.93	0.99	1.23	1.54
VRC13.01		0.02	0.04	0.17	0.38	0.68	1.01	1.07	1.31	1.61
b12		-0.19	0.06	0.23	0.44	0.69	0.97	0.96	1.21	1.42
CH103		-2.15	0.05	0.23	0.52	0.86	1.18	1.24	1.46	1.72
VRC16.01		-2.23	0.03	0.16	0.36	0.61	0.94	1.01	1.31	1.68
1-18		0.24	0.03	0.18	0.39	0.63	0.90	0.95	1.19	1.50
VRC-PG05	Silent face	7.62	0.09	0.38	0.72	1.09	1.41	1.48	1.75	2.15
SF12		6.36	0.09	0.32	0.64	1.06	1.56	1.61	2.07	2.57
PGT151	Fusion peptide	-0.81	0.05	0.19	0.38	0.62	0.87	0.91	1.15	1.49
VRC34.01		-0.76	0.02	0.09	0.20	0.36	0.51	0.54	1.15	0.79
35O22	Subunit interface	3.76	0.04	0.16	0.32	0.50	0.66	0.69	0.83	1.02
8ANC195		-0.02	0.08	0.24	0..45	0.69	0.89	0.93	1.07	1.27

Supplementary Table 3 | Cryo-EM data collection and refinement statistics.

	2909 Fab in complex with CAP256.wk34.c80 SOSIP.RnS2 N160K HIV-1 Env trimer and 3BNC117 Fab
**EMDB ID**	23589
**PDB ID**	7LY9
Data Collection	
Microscope	FEI Titan Krios
Voltage (kV)	300
Electron dose ($e^-/Å^2$)	66.88
Detector	Gatan K2 Summit
Pixel Size (Å)	1.096
Defocus Range (μm)	-1.0 to -2.5
Magnification	105000
Reconstruction	
Software	cryoSparcV2.15
Particles	35,406
Symmetry	C1
Box size (pix)	400
Resolution (Å) ($FSC_{0.143}$)	3.91
Refinement	
Software	Phenix 1.18
Protein residues	2538
Chimera CC	0.81
EMRinger Score	1.44
R.m.s. deviations	
Bond lengths (Å)	0.005
Bond angles (°)	0.976
Validation	
Molprobity score	1.59
Clash score	4.07
Favored rotamers (%)	99.2
Ramachandran	
Favored regions (%)	94.2
Disallowed regions (%)	0.04

Supplementary Table 4 | Epitope surface area and other structural properties of antibody PGT145 and 2909.

(a) The epitope residue surface area of PGT145 (left) and 2909 (right).

(b) The properties describing the difference between BG505 WT and BG505 N160K of antibody PGT145 and 2909.

a

PGT145 epitope residue number (chain ID)	Buried surface area (Å^2)
130 (A)	10.8
160 (A)	46.3
166 (A)	6.6
167 (A)	10.5
168 (A)	26.8
169 (A)	144.9
171 (A)	36.3
121 (C)	0.3
123 (C)	0.8
130 (C)	4.2
160 (C)	33
162 (C)	7.4
166 (C)	3.1
167 (C)	32.4
168 (C)	4.4
169 (C)	123.5
190 (C)	23.2
121 (E)	0.7
123 (E)	0.5
160 (E)	45.1
166 (E)	6.8
167 (E)	45.5
168 (E)	27
169 (E)	149.7
171 (E)	0.5

2909 epitope residue number (chain ID)	Buried surface area (Å^2)
123 (A)	1.8
130 (A)	36.7
160 (A)	16.5
166 (A)	1.6
167 (A)	29.5
168 (A)	77.7
169 (A)	105.9
171 (A)	154.7
184 (A)	18.2
185 (A)	61.7
185A (A)	15.2
185B (A)	161.5
185D (A)	40.5
123 (C)	1.9
130 (C)	83.3
132 (C)	5.1
158 (C)	2.8
160 (C)	13.7
166 (C)	1.1
167 (C)	25.4
168 (C)	85.5
169 (C)	107.8
170 (C)	55.8
171 (C)	209.8
173 (C)	3.8
185B (C)	72.6
185D (C)	30.8
123 (E)	2.1
130 (E)	69
158 (E)	0.01
160 (E)	16.2
166 (E)	1.3
167 (E)	30.7
168 (E)	65.8
169 (E)	100.8
170 (E)	16.1
171 (E)	179.2
185B (E)	47.4
185D (E)	34.2

b

**Antibody**	**AFAD relative to mean (Å)**	**Average number of glycan atoms in WT**	**Average number of glycan atoms in N160K**	**Difference of average number of glycan atoms between WT and N160K (count)**	**Buried surface area (Å^2)**	**Difference of average epitope-glycan coverage between WT and N160K (count/Å^2)**
PGT145	15.23	2303.34	1910.55	392.79	790.41	0.50
2909	1.86	3003.76	2679.84	323.92	1983.46	0.16

Supplementary Table 5 | PDB code for antibodies plotted in Supplementary Figures 3, 4 and 5.

Antibody	PDB code
VRC26.25	6VTT
PGT145	5V8L
PG9	3U2S
VRC38.01	5VGJ
2909	7LY9
PGT121-3H+109L	5CEZ
PGT128	5ACO
PGT135	4JM2
2G12	6E5P
HJ16	4YE4
IOMA	5T3Z
CH235.09	5F9O
VRC01	5FYJ
VRC13.01	4YDJ
b12	5VN8
CH103	4JAN
VRC16.01	4YDK
1-18	6UDJ
VRC-PG05	6BF4
SF12	6OKP
PGT151	5FUU
VRC34.01	5I8H
35O22	5CEZ
8ANC195	5CJX

Supplementary Table 6 | Antibody 2909, 3BNC117, PGT145 and 2G12 codon optimized DNA sequences.

Antibody expression construct	Codon-optimized DNA sequences of antibody variable region
2909 Heavy chain	GAGGTGCAGCTGGTTGAGTCTGGCGGAAATGTGGTTCAGCCTGGCGGCAGCCTGAGACTGTCTTGT<br>ACAGCCAGCGGCTTCAGCTTCGACGACAGCACAATGCACTGGGTCCGACAGGCCCCTGGAAAAGGA<br>CTGCAATGGGTGTCCCTGATCAGCTGGAATGGCGGCAGAACCTACTACGCCGACAGCGTGAAGGGC<br>AGATTCACCATCAGCCGGGACAACAGCAAGAACAGCCTGTACCTGCAGATGAACTCCCTGAAAACC<br>GAGGATACCGCCTTCTACTTCTGCGCCAAGGACAAGGGCGACAGCGACTACGACTACAACCTGGGC<br>TACAGCTACTTCTACTACATGGACGGCTGGGGCAAGGGCACCACCGTGACAGTTTCTTCT
2909 Light chain	AGCTACGTGCTGACACAGCCTCCATCCGTGTCTGTGGCCCCTGGAAAGACCGCCAGAATCACATGC<br>GGCGGCAACAATATCGCCAACAAGAACGTGCACTGGTATCAGCAGAAGCCCGGACAGGCTCCTGTG<br>CTGGTCATCTACTACGACGACGACAGACCCAGCGGCATCCCCGATAGATTCAGCGGCAGCAACAGC<br>GGCAATACCGCCACACTGACCATCAGCAGAGTGGAAGCTGGCGACGAGGCCGACTACTACTGCCAA<br>GTGTGGGACAGCAACTCCGATCACGTGGTGTTTGGCGGCGGAACACAACTG
3BNC117 Heavy chain	CAGGTCCAATTGTTACAGTCTGGGGCAGCGGTGACGAAGCCCGGGGCCTCAGTGAGAGTCTCCTGC<br>GAGGCTTCTGGATACAACATTCGTGACTACTTTATTCATTGGTGGCGACAGGCCCCAGGACAGGGCC<br>TTCAGTGGGTGGGATGGATCAATCCTAAGACAGGTCAGCCAAACAATCCTCGTCAATTTCAGGGTAG<br>AGTCAGTCTGACTCGACACGCGTCGTGGGACTTTGACACATTTTCCTTTTACATGGACCTGAAGGCA<br>CTAAGATCGGACGACACGGCCGTTTATTTCTGTGCGCGACAGCGCAGCGACTATTGGGATTTCGAC<br>GTCTGGGGCAGTGGAACCCAGGTCACTGTCTCGTCA
3BNC117 Light chain	TCAGACATCCAGATGACCCAGTCTCCATCCTCCCTGTCTGCCTCTGTGGGAGATACCGTCACTATCA<br>CTTGCCAGGCAAACGGCTACTTAAATTGGTATCAACAGAGGCGAGGGAAAGCCCCAAAACTCCTGAT<br>CTACGATGGGTCCAAATTGGAAAGAGGGGTCCCATCAAGGTTCAGTGGAAGAAGATGGGGGCAAGA<br>ATATAATCTGACCATCAACAATCTGCAGCCCGAAGACATTGCAACATATTTTTGTCAAGTGTATGAGTT<br>TGTCGTCCCTGGGACCAGACTGGATTTGAAACGT
PGT145 Heavy chain	CAGGCTAGCACCATGGATTGGATTTGGCGCATTTTGTTTTTGGTTGCCGCTGCCACCAGCGCCCATT<br>CTCAAGTTCAGTTGGTTCAGTCTGGTGCCGAAGTTAAAAAACCGGGTTCTTCTGTTAAAGTTAGCTGT<br>AAAGCCAGCGGTAATTCTTTTTCTAATCATGATGTTCATTGGGTTCGCCAGGCCACCGGCCAAGGCT<br>TGGAATGGATGGGTTGGATGAGCCATGAAGGCGATAAAACCGGCTTGGCTCAAAAATTTCAGGGCC<br>GTGTTACCATTACCCGCGATAGCGGTGCCTCTACCGTTTATATGGAATTGCGCGGTTTGACCGCTGA<br>TGATACCGCCATCTATTATTGTTTGACCGGCTCTAAACATCGTTTGCGCGATTATTTTCTTTATAATGA<br>ATATGGCCCGAACTATGAAGAATGGGGTGACTATTTGGCTACCTTGGATGTTTGGGGTCATGGTACC<br>GCTGTTACCGTTTCTTCT
PGT145 Light chain	GAGGTCGTGATCACACAGTCGCCCCTGTTTCTGCCCGTGACGCCCGGAGAGGCAGCGTCCTTGTCA<br>TGCAAATGTAGCCACAGCCTGCAACATTCAACTGGCGCCAACTATCTTGCATGGTACTTGCAGCGGC<br>CAGGTCAGACACCGAGACTCCTGATCCATCTTGCGACCCACCGAGCCTCCGGGGTCCCTGATCGCT<br>TCTCGGGATCGGGTAGCGGAACAGACTTCACCCTTAAGATTTCAAGGGTCGAATCCGACGATGTGG<br>GGACGTATTACTGCATGCAAGGTTTGCACTCGCCTTGGACTTTTGGGCAGGGAACGAAGGTAGAAAT<br>TAAGCGG
2G12 Heavy chain	GAGGTGCAGCTGGTCGAGTCTGGCGGCGGACTGGTCAAGGCAGGCGGGTCTCTGATTCTGAGTTG<br>TGGGGTCTCCAATTTTCGGATTTCCGCTCACACCATGAACTGGGTGCGGAGAGTCCCCGGAGGAGG<br>ACTGGAGTGGGTGGCCTCCATCTCTACTAGCTCCACCTACCGGGACTATGCAGATGCCGTGAAGGG<br>GAGGTTCACAGTCTCTCGCGACGATCTGGAGGACTTTGTGTACCTGCAGATGCATAAGATGCGGGT<br>CGAAGATACTGCTATCTACTATTGCGCACGAAAAGGCTCAGACAGACTGAGCGACAATGATCCTTTC<br>GATGCCTGGGGACCAGGCACAGTGGTCACTGTGAGCCCC
2G12 Light chain	GTCGTGATGACCCAGAGTCCAAGCACCCTGTCCGCAAGTGTCGGGGATACCATTACCATTACCTGTC<br>GGGCAAGCCAGTCTATTGAAACCTGGCTGGCCTGGTATCAGCAGAAGCCAGGGAAAGCTCCCAAGC<br>TGCTGATCTATAAAGCATCTACACTGAAGACTGGAGTGCCCAGTCGATTCTCAGGCAGCGGGTCCG<br>GAACCGAGTTTACCCTGACAATTAGCGGCCTGCAGTTCGACGATTTTGCTACATACCACTGCCAGCA<br>TTACGCAGGGTATAGCGCAACCTTCGGACAGGGGACACGGGTGGAGATCAAAAGA
