# Supplementary material for: Extended antibody-framework-to-antigen distance observed exclusively with broad HIV-1-neutralizing antibodies recognizing glycan-dense surfaces
Source: Nat Commun. 2021 Nov 9;12:6470. doi: 10.1038/s41467-021-26579-z (PMC8578620; doi:10.1038/s41467-021-26579-z)
Supplement: Supplementary file 2 — Description of Additional Supplementary Files [file 41467_2021_26579_MOESM2_ESM.pdf]

Description of Additional Supplementary Files

Supplementary Data 1 | Antibody neutralization evaluated on 208 HIV-1 isolates.
