# Supplementary material for: Extended antibody-framework-to-antigen distance observed exclusively with broad HIV-1-neutralizing antibodies recognizing glycan-dense surfaces
Source: Nat Commun. 2021 Nov 9;12:6470. doi: 10.1038/s41467-021-26579-z (PMC8578620; doi:10.1038/s41467-021-26579-z)
Supplement: Supplementary file 4 — Reporting summary [file 41467_2021_26579_MOESM4_ESM.pdf]

nature research

Corresponding author(s): Gwo-Yu Chuang and Peter D. Kwong

Last updated by author(s): Sep 8, 2021

Reporting Summary

Nature Research wishes to improve the reproducibility of the work that we publish. This form provides structure for consistency and transparency in reporting. For further information on Nature Research policies, see our Editorial Policies and the Editorial Policy Checklist.

Statistics

For all statistical analyses, confirm that the following items are present in the figure legend, table legend, main text, or Methods section.

n/a	Confirmed	
☐	☒	The exact sample size (*n*) for each experimental group/condition, given as a discrete number and unit of measurement
☐	☒	A statement on whether measurements were taken from distinct samples or whether the same sample was measured repeatedly
☐	☒	The statistical test(s) used AND whether they are one- or two-sided<br>*Only common tests should be described solely by name; describe more complex techniques in the Methods section.*
☒	☐	A description of all covariates tested
☒	☐	A description of any assumptions or corrections, such as tests of normality and adjustment for multiple comparisons
☐	☒	A full description of the statistical parameters including central tendency (e.g. means) or other basic estimates (e.g. regression coefficient) AND variation (e.g. standard deviation) or associated estimates of uncertainty (e.g. confidence intervals)
☐	☒	For null hypothesis testing, the test statistic (e.g. *F*, *t*, *r*) with confidence intervals, effect sizes, degrees of freedom and *P* value noted<br>*Give P values as exact values whenever suitable.*
☒	☐	For Bayesian analysis, information on the choice of priors and Markov chain Monte Carlo settings
☒	☐	For hierarchical and complex designs, identification of the appropriate level for tests and full reporting of outcomes
☒	☐	Estimates of effect sizes (e.g. Cohen's *d*, Pearson's *r*), indicating how they were calculated

Our web collection on statistics for biologists contains articles on many of the points above.

Software and code

Policy information about availability of computer code

Data collection	NAMD2.13 was used for molecular dynamics simulations. Leginon v3.5 was used to collect cryoEM data. YASARA20.12.24 was for homology modeling, RATTLE algorithm was used to constraints length of bonds during molecular dynamics simulations. Glycosylator was used to glycosylate proteins. The in-house program, GLYCO is available at https://github.com/myungjinlee/GLYCO
Data analysis	Python3.7 was used for coding. FreeSASA2.0.3 was used to calculate buried surface area. CD-HIT4.6.8 was for removing redundant antibodies in the dataset. IGBLAST server was used to examine somatic hypermutation. ForteBio Data Analysis 11.0 was used to analyze Octet binding measurements. CryoSPARC v2.15 and Phenix v1.18 were used to process cryoEM data and refine the PDB. PyMOL v2.4.2 and ChimeraX v1.1.1 were for visualization.

For manuscripts utilizing custom algorithms or software that are central to the research but not yet described in published literature, software must be made available to editors and reviewers. We strongly encourage code deposition in a community repository (e.g. GitHub). See the Nature Research guidelines for submitting code & software for further information.

Data

Policy information about availability of data

All manuscripts must include a data availability statement. This statement should provide the following information, where applicable:

- Accession codes, unique identifiers, or web links for publicly available datasets
- A list of figures that have associated raw data
- A description of any restrictions on data availability

EMDB: EMD-23589
PDB: 7LY9 (https://doi.org/10.2210/pdb7LY9/pdb)
Antibody dataset: SabDab (http://opig.stats.ox.ac.uk/webapps/newsabdab/sabdab)

Field-specific reporting

Please select the one below that is the best fit for your research. If you are not sure, read the appropriate sections before making your selection.

- [x] Life sciences
- [ ] Behavioural & social sciences
- [ ] Ecological, evolutionary & environmental sciences

For a reference copy of the document with all sections, see nature.com/documents/nr-reporting-summary-flat.pdf

Life sciences study design

All studies must disclose on these points even when the disclosure is negative.

Sample size	1879 antibody-complexes of PDB for the Antibody-Framework-to-Antigen Distance (AFAD) and 23 broadly neutralizing antibodies of HIV-1 for correlations between AFAD and antigen properties were chosen as sample sizes. This includes all available non-redundant structures in PDB database in SAbDab, and all 23 HIV-1 broadly neutralizing antibodies within the 1879 PDB sample. Since non-redundant all PDB structures were counted, the size of sample is sufficient.
Data exclusions	No data were excluded.
Replication	Neutralization data were from a single run or reported as the geometric mean of two runs. When two runs were performed, IC50 and IC80 values were generally within 2-fold of each other, which is considered successful replication in this assay (Sarzotti-Kelsoe, J Imm Methods, 2014). Binding assays were performed at least twice to verify antibody binding.
Randomization	Randomization was not performed because the entire PDB was analyzed.
Blinding	Blinding was not applied. Experimental group allocation and issues of selection bias did not impact this study.

Reporting for specific materials, systems and methods

We require information from authors about some types of materials, experimental systems and methods used in many studies. Here, indicate whether each material, system or method listed is relevant to your study. If you are not sure if a list item applies to your research, read the appropriate section before selecting a response.

Materials & experimental systems

n/a	Involved in the study
[ ]	[x] Antibodies
[ ]	[x] Eukaryotic cell lines
[x]	[ ] Palaeontology and archaeology
[x]	[ ] Animals and other organisms
[x]	[ ] Human research participants
[x]	[ ] Clinical data
[x]	[ ] Dual use research of concern

Methods

n/a	Involved in the study
[x]	[ ] ChIP-seq
[x]	[ ] Flow cytometry
[x]	[ ] MRI-based neuroimaging

Antibodies

Antibodies used	Recombinant human monoclonal antibodies 2G12, 2909, 3BNC117, and PGT145 were produced in-house by transient transfection in Expi293F cells. Antibodies were used either directly from culture supernatant or as purified proteins at concentrations specified in the methods.
Validation	Antibodies were validated by antigen-binding and neutralization analyses, as described in cited publications or data shown in the manuscript.

Eukaryotic cell lines

Policy information about cell lines

Cell line source(s)	Expi293F Cells is a product of Thermo Fisher Scientific, Catalog number: A14527. FreeStyle 293-F Cells (Thermo Fisher catalog number R79007). TZM-bl cells, also called JC53-bl (clone 13), were obtained from Dr. John C. Kappes, Dr. Xiaoyun Wu and Tranzyme Inc. via the NIH AIDS Research and Reference Reagent Program, Division of AIDS, NIAID, NIH.
Authentication	None of the cell lines used were authenticated.
Mycoplasma contamination	The cell lines were not tested for mycoplasma contamination.

Commonly misidentified lines<br>(See ICLAC register)	No commonly misidentified cell lines were used in the study.
